# Mechanical Strength Enhancement of 3D Printed Acrylonitrile Butadiene Styrene Polymer Components Using Neural Network Optimization Algorithm

**DOI:** 10.3390/polym12102250

**Published:** 2020-09-30

**Authors:** Jasgurpreet Singh Chohan, Nitin Mittal, Raman Kumar, Sandeep Singh, Shubham Sharma, Jujhar Singh, Kalagadda Venkateswara Rao, Mozammel Mia, Danil Yurievich Pimenov, Shashi Prakash Dwivedi

**Affiliations:** 1 University Centre for Research and Development, Chandigarh University, Mohali-140413, India; jaskhera@gmail.com (J.S.C.); mittal.nitin84@gmail.com (N.M.); ramankakkar@gmail.com (R.K.); drsandeep1786@gmail.com (S.S.); 2 Department of Mechanical Engineering, IKG Punjab Technical University, Kapurthala-144603, India or shubhamsharmacsirclri@gmail.com (S.S.); jujharsing2085@gmail.com (J.S.); 3 Center for Nanoscience and Technology, Institute of Science and Technology, Jawaharlal Nehru Technological University Hyderabad, Telangana State 500085, India; kalagadda2003@gmail.com; 4 Department of Mechanical Engineering, Imperial College London, Exhibition Rd., London SW7 2AZ, UK; 5 Department of Automated Mechanical Engineering, South Ural State University, Lenin Prosp. 76, 454080 Chelyabinsk, Russia; danil_u@rambler.ru; 6 Department of Mechanical Engineering, G.L. Bajaj Institute of Technology and Management, Greater Noida 201308, India; spdglb@gmail.com

**Keywords:** optimization, neural network algorithm, fused filament fabrication, mechanical strength, simulation

## Abstract

Fused filament fabrication (FFF), a portable, clean, low cost and flexible 3D printing technique, finds enormous applications in different sectors. The process has the ability to create ready to use tailor-made products within a few hours, and acrylonitrile butadiene styrene (ABS) is extensively employed in FFF due to high impact resistance and toughness. However, this technology has certain inherent process limitations, such as poor mechanical strength and surface finish, which can be improved by optimizing the process parameters. As the results of optimization studies primarily depend upon the efficiency of the mathematical tools, in this work, an attempt is made to investigate a novel optimization tool. This paper illustrates an optimization study of process parameters of FFF using neural network algorithm (NNA) based optimization to determine the tensile strength, flexural strength and impact strength of ABS parts. The study also compares the efficacy of NNA over conventional optimization tools. The advanced optimization successfully optimizes the process parameters of FFF and predicts maximum mechanical properties at the suggested parameter settings.

## 1. Introduction

Globalization has intensified the market scenario of manufacturing units due to rapidly changing customer demands, competition among peers and requirements for high quality and reliable products. The demand for low cost and customized products has led to a paradigm shift from traditional manufacturing techniques to additive manufacturing technologies by industries [1]. Additive manufacturing is a collection of non-conventional techniques that follow the principle of layer by layer manufacturing in contrast to traditional subtractive and joining techniques [2]. The last decade witnessed a marginal increase in the development and market share of various additive manufacturing techniques due to their implementation in medical, aerospace, automobile, military and ornamental industries [3]. Additive manufacturing enables direct fabrication of a product from three-dimensional computer-aided design (CAD) data through successive layer stacking of suitable material. These techniques are also popularly referred to as 3D printing, rapid prototyping, solid freeform fabrication, e-manufacturing and digital fabrication. There are more than thirty different additive manufacturing techniques available, which differ on fabrication principle, materials used, accuracy, bed size, part strength and applicability; however, fused filament fabrication (FFF) is the most recognized [4].

**Figure 1:**
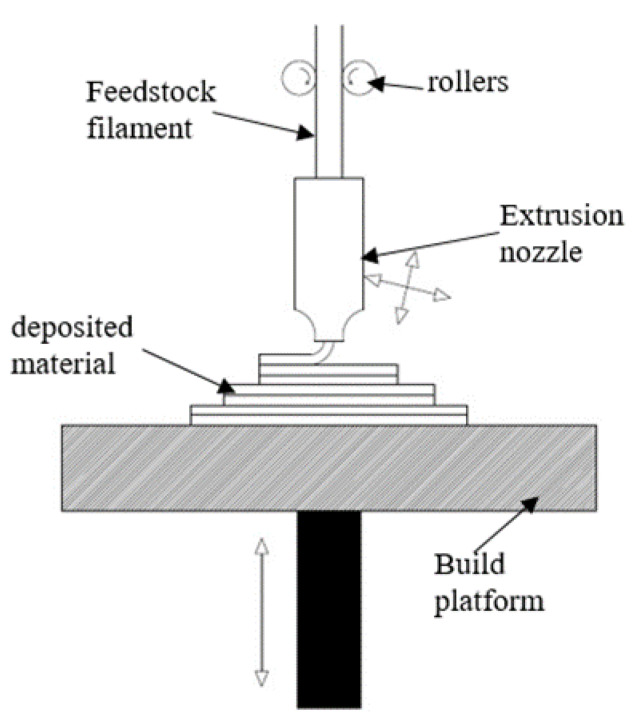
Schematic of the fused filament fabrication (FFF) process used for 3D printing of thermoplastics.

FFF has acquired considerable attention from researchers, material scientists, innovators and medical practitioners due to the unique advantages of this technique, such as material flexibility, minimum environmental degradation, low cost, portability and higher accuracy [5]. FFF uses CAD data as input, which is transferred to slicing software for tool path generation. These tool paths are coded instructions that control the three-dimensional motion of the nozzle head and build platforms to create the product within a few hours. The raw material used is a thin wire of thermoplastic material with a lower melting point and zero toxicity. This wire is pushed by rollers into the nozzle head, where heaters convert it into semi-molten filament. Finally, the material is deposited by a nozzle moving in X and Y direction on the build platform, as shown in Figure 1. As one layer is deposited, the build platform moves downwards (in Z direction), and the next layer of material is deposited. The process is repeated until the desired part is achieved. Sometimes, another filament of washable material is used to support the overhanging part, which is easily washed away after fabrication [6]. 

FFF supplies customized products with the minimum lead time and manufacturing cost, but the mechanical strength of the part is always a matter of interest for researchers as considerable variation in mechanical properties is experienced due to variation in design [7]. Moreover, issues related to the lower mechanical strength of FFF parts may hinder the usability of these products for certain applications. Thus, there is always a requirement for intelligent optimization tools for the prediction and maximization of the mechanical strength of FFF parts. There are several input parameters of FFF technology, which have a significant impact on the tensile strength, compressive strength, flexural and impact strength of FFF parts [5]. Although numerous studies have been conducted for optimization of process parameters of FFF, recent studies have focused on the development of conventional mathematical tools and algorithms which can optimize and forecast the mechanical strength of FFF parts. 

Hambali et al. [2] tested the deformation behavior of acrylonitrile butadiene styrene (ABS) material parts made by FFF technology. The parts were fabricated at different orientations, and it was found that the maximum strength occurred at a 45° angle. The study was validated by finite element analysis, which predicted the mechanical strength with 95% accuracy. Other studies also indicated the significant impact of orientation angle [3] and infill density [8] on the mechanical strength of FFF parts. Ahn et al. [7] investigated the anisotropic behavior of FFF parts and found that the air gap and raster orientation significantly affected the tensile strength of FFF parts. The cross raster structure (0°/90°) yielded the maximum tensile strength due to its directional stability, and it was suggested that a negative air gap be maintained for higher strength and stiffness. In addition to conventional optimization studies, researchers have used mathematical models and algorithms for the analysis of process parameters. Multi-criteria genetic algorithm was used for the optimization and prediction of the surface finish and manufacturing time of FFF parts [4] and aluminum-based metal matrix composites [9]. The teaching–learning based optimization tool was utilized by Rao and Rai [1] to enhance the sliding wear resistance and strength of FFF parts; the experimental data was compared with genetic algorithm and advanced quantum-behaved particle swarm optimization algorithm, which found strong correlation between experimental and computational data. Rayegani and Onwubolu [5] developed a relationship between different factors, such as part orientation, raster angle, raster width and air gap, with tensile strength of FFF parts using differential evolution techniques along with the group method of data handling (GMDH). It was deduced that deposition and raster angle defined the FFF part strength. Moreover, the fine-tuning of input parameters was performed using advanced tools to achieve maximum mechanical strength. Natarajan et al. [10] also implemented a modified teaching–learning based optimization method (NSMTLBO), referred to as non-dominated sorting type, to acquire maximum finish and minimum material removal rate.

In another study, a hybrid version of particle swarm technique was applied to FFF input parameters, such as layer thickness, deposition angle, material type and infill strategy, and for improving the surface finish, hardness, flexural strength and tensile strength in combination with bacterial foraging optimization [11]. The tool yielded promising results as higher strength (more than 7%) was achieved using the hybrid optimization technique as compared to conventional algorithms. Recent studies have reported the impact of orientation angle and infill percentage in mechanical properties of ABS samples [12]. Moreover, in the case of polylactic acid material, the anisotropy of samples had a significant impact on tensile strength as compared to the orientation angle [13]. In addition to optimization tools, some advanced post-processing techniques, such as annealing and ultrasound treatment, have been recommended for strength enhancement [14].

Zhang et al. [15] found that residual stress and porosity were directly proportional to printing speed during the fabrication of virgin and composite ABS structures. Moreover, the raster angle of ±45° manifested lesser shrinkage and porosity, which further influenced dimensional accuracy and mechanical strength, respectively. Similarly, Rankouhi et al. [16] found a major impact of layer thickness and raster orientation on the mechanical strength of FFF parts, with a reduction in layer thickness and 0° raster angle strengthening the ABS structures. Another study reported the maximum impact strength of the ABS part at 0° orientation angle [17]. Christiyan et al. [18] evaluated the mechanical strength of magnesium silicate reinforced ABS composites and found maximum tensile and flexural strength at minimum values of layer thickness and printing speed. The increase in infill density also enhanced the tensile behavior of ABS test samples [19]. The flexural and impact strength of ABS samples under standard conditions was maximum with thinner layer thickness and 0° deposition angle [20,21].

The previous studies reported the impact of different input parameters on the mechanical properties of ABS parts made by FFF technology. Although smaller layer thickness is uniformly recommended by previous literature, the recommended settings of the raster angle, orientation angle and air gap are different for each study. These issues are due to conventional optimization algorithms and mathematical modeling tools used for forecasting optimum parameters, which yield conflicting results. Moreover, limited studies are performed where advanced optimization algorithms are used for prediction of the best parametric settings for mechanical strength enhancement. In the present work, the efficacy of neural network algorithm (NNA) is tested to solve the mechanical strength issues of FFF parts. As the introduction of advanced optimization algorithms have yielded significant improvements compared to previous studies performed by regular algorithms, investigating the optimization problems using this optimization and simulation technique is necessary.

In the next section, a detailed methodology of the formulation and implementation of NNA is elaborated, along with a selection of objective functions and process parameters. The third section presents a comparative analysis of different algorithms and a discussion of the simulation results. Finally, the fourth section includes confirmatory experiments that are conducted to validate the predicted results. 

## 2. Materials and Methods

### 2.1. Neural Network Optimization Algorithm

Sadollah et al. [22] developed a metaheuristics based on neural networks (NNs) named neural network algorithm (NNA). The authors found that a metaheuristic optimization algorithm could be used to model artificial NNs (ANNs) to solve optimization problems. NNA was developed using the ANNs framework to solve optimization problems. NNA is a parameter-free optimization algorithm where users do not require any algorithm parameters to be adjusted.

ANNs iteratively update the ANN’s weights $w_{ij}$ to minimize the mean square error and map the input information according to the target information required at its output. ANNs are designed to produce new alternatives, where the population’s best search agent is considered as the target and all searching agents seek the solution through algorithm processes.

NNA draws on the concept and structure of ANN. NNA begins within a search space with an initial population of randomly generated solutions. Each individual in the population is referred to as a “pattern solution”, which is a vector of 1 × D, representing the input data of the NNA.

$$PatternSolution_{i}=\left[x_{i,1},x_{i,2},x_{i,3},\ldots ,x_{i,D}\right].$$

To start the NNA optimization algorithm, within the boundaries of the search space, a pattern solution matrix X with size $N_{pop}\times D$ is generated randomly, given by:

(1) $$X=\left[\begin{array}{c} X_{1}\\ X_{2}\\ .\\ .\\ .\\ X_{N_{pop}} \end{array}\right]=\left[\begin{array}{c} \begin{array}{ccc} x_{1,1} & x_{1,2} & x_{1,D} \end{array}\\ \begin{array}{ccc} x_{2,1} & x_{2,2} & x_{2,D} \end{array}\\ \begin{array}{ccc} . & . & . \end{array}\\ \begin{array}{ccc} . & . & . \end{array}\\ \begin{array}{ccc} . & . & . \end{array}\\ \begin{array}{ccc} x_{N_{pop},1} & x_{N_{pop},2} & x_{N_{pop},D} \end{array} \end{array}\right]$$

where:

$$x_{ij}=LB_{j}+rand\left(UB_{j}-LB_{j}\right),i=1,2,\ldots ,N_{pop},j=1,2,\ldots ,D$$

where LB and UB represent the search space’s lower and upper limits.

Similar to ANNs, every pattern solution $X_{i}$ in NNA will have its corresponding $W_{i}$ weight, where:

$$W_{i}={\left[w_{i,1},w_{i,2},w_{i,3},\ldots ,w_{i,N_{pop}}\right]}^{T}$$

The weights array W is given by:

(2) $$W=\left[W_{1},W_{2},\ldots ,W_{i},\ldots ,W_{N_{pop}}\right]=\left[\begin{array}{c} \begin{array}{ccc} w_{11} & w_{i1} & \ldots w_{N_{pop}1} \end{array}\\ \begin{array}{ccc} w_{12} & w_{i2} & \ldots w_{N_{pop}2} \end{array}\\ \begin{array}{ccc} . & . & . \end{array}\\ \begin{array}{ccc} . & . & . \end{array}\\ \begin{array}{ccc} . & . & . \end{array}\\ \begin{array}{ccc} w_{1N_{pop}} & w_{iN_{pop},2} & \ldots w_{N_{pop}N_{pop}} \end{array} \end{array}\right]$$

where W represents a matrix of a $\left(N_{pop}\times N_{pop}\right)$ randomly distributed number in [0, 1], used to generate new candidate solutions.

The initial weights in NNA are randomly generated and the network changes their weights according to the network error propagation as the iteration increases. The weight values are limited, as the overall weight of this pattern solution must not exceed one and the solution for the weight pattern is defined as:

(3) $$w_{ij}\in U\left(0,1\right),i,j=1,2,3,\ldots ,N_{pop}$$

(4) $$\overset{N_{pop}}{\underset{j=1}{\sum }}w_{ij}=1,i=1,2,3,\ldots ,N_{pop}$$

Such weight-value constraints are used to regulate bias movement and to generate new pattern solutions. NNA will get trapped in the local optimum without these constraints. The fitness $f_{i}$ of each solution is determined using the corresponding pattern solution $X_{i}$ by evaluating the objective function $f_{obj}$.

(5) $$f_{i}=f_{obj}(X_{i})=f_{obj}\left(x_{i1},x_{i2},x_{i3},\ldots ,x_{iD}\right),i=1,2,3,\ldots ,N_{pop}$$

After fitness calculation for all pattern solutions, the pattern solution with the best fitness is considered as the target solution, with a target location $X^{Target}$, target fitness $F^{Target}$ and target weight $W^{Target}$. The new pattern solution is created using the weight summation technique used in ANNs as follows:

(6) $$X_{j}^{New}\left(k+1\right)=\overset{N_{pop}}{\underset{i=1}{\sum }}w_{ij}\left(k\right).X_{i}\left(k\right),j=1,2,3,\ldots ,N_{pop}$$

(7) $$X_{i}\left(k+1\right)=X_{i}\left(k\right)+X_{i}^{New}\left(k+1\right),i=1,2,3,\ldots ,N_{pop}$$

where  k is an iteration index.

After the generation of the new pattern solutions, the weight matrix is also modified using the equation:

(8) $$W_{i}^{Updated}\left(k+1\right)=W_{i}\left(k\right)+2.rand.\left(W^{Target}\left(k\right)-W_{i}\left(k\right),\right),i=1,2,3,\ldots ,N_{pop}$$

where the constraints (3) and (4) during the optimization process must be satisfied.

A bias operator is used to better explore the search space by adjusting a fixed range of pattern solutions created in the new $X_{i}\left(k+1\right)$ population as well as using the updated weight matrix $W_{i}^{Updated}\left(k+1\right)$. This operator helps the algorithm prevent premature convergence by changing the few individuals in the population to investigate certain positions in the search space that were not explored yet.

**Figure 2:**
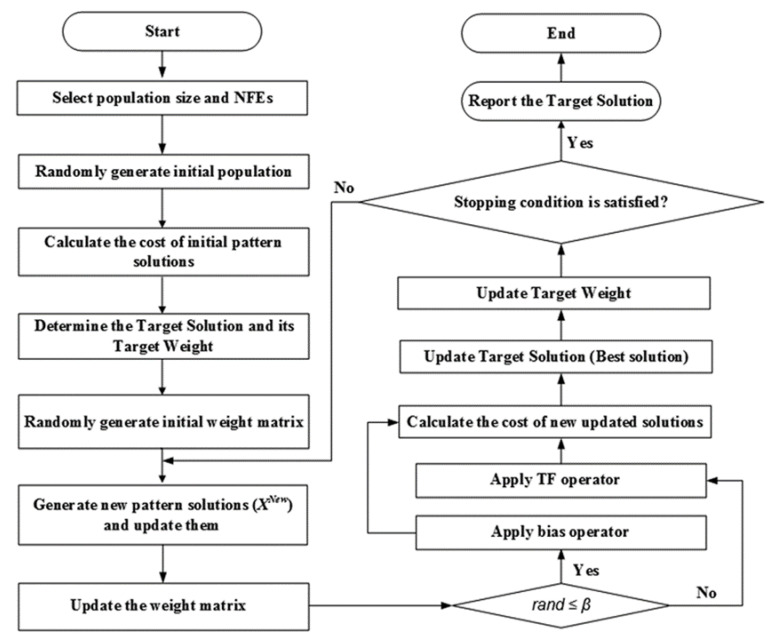
Flowchart adopted during implementation neural network algorithm (NNA) (NFE, number of function evaluations; TF, transfer function).

An adjustment factor $\beta_{NNA}$ is used, using the bias operator, to determine the proportion of pattern solutions to be transformed. The value of $\beta_{NNA}$ is set to 1 initially, which means all the entities in the population are biased. At each iteration, the $\beta_{NNA}$ value will be reduced adaptively using any possible reduction technique, such as:

(9) $$\beta_{NNA}\left(k+1\right)=1-\left(\frac{k}{Max\_iteration}\right),k=1,2,3,\ldots ,Max\_iteration$$

(10) $$\beta_{NNA}\left(k+1\right)=\beta_{NNA}\left(k\right).\alpha_{NNA},k=1,2,3,\ldots ,Max\_iteration$$

where $\alpha_{NNA}$ is a positive number slightly smaller than 1. The flowchart of the NNA process is given in Figure 2.

Reduction in the value of $\;\beta_{NNA}$ is done to enhance the algorithm’s exploitation during the final iterations. The transfer function operator is used in NNA (unlike ANNs) to generate better-quality solutions using the following equation:

(11) $$X_{i}^{*}\left(k+1\right)=TF\left(X_{i}\left(k+1\right)\right)=X_{i}\left(k+1\right)+2.rand.\left(X^{Target}\left(k\right)-X_{i}\left(k+1\right)\right)\;i=1,2,3,\ldots ,N_{pop}$$

In NNA, the bias operator has more chances to produce a new pattern solution at early iterations, meaning that there are more possibilities to find unvisited pattern solutions. As the number of iterations increases, the probability of applying the bias operator decreases. The transfer function (TF) operator has more chances of enhancing the exploitation, particularly at the final iterations.

The generation of a new updated solution in NNA does not depend solely on the preceding value of that solution but also depends on all the mathematically defined population (known as a dynamic optimization model), as follows:

(12) $$X_{i}\left(k+1\right)=f\left(X_{i}\left(k\right),X\left(k\right)\right),i=1,2,3,\ldots ,N_{pop}$$

### 2.2. Selection of Process Parameters

The materials selected for the present study were ABS commercial-grade P400 (Supplier: Stratasys Ltd., Eden Prairie, Minnesota, Inc., USA), which was manufactured by the polymerization of styrene (42–60%) and acrylonitrile (18–35%) in an atmosphere of butadiene (6–30%). The criss-cross bonding of long-chained butadiene with shorter styrene-acrylonitrile chains yields excellent toughness and heat resistance. The filament also contained mineral oil, tallow and wax in small concentrations (0–2%), as reported in the manufacturer’s datasheet [23]. The output of the case study performed by Panda et al. [24] was considered to identify the optimum parameter settings of the FFF process to enhance the mechanical properties of the test samples. The five input parameters considered for the present study and their units were as follows:

$x_{1}$ Layer thickness in mm.
$x_{2}$ Orientation in degrees.
$x_{3}$ Raster angle in degrees.
$x_{4}$ Raster width in mm.
$x_{5}$ Air gap in mm.

The major reason for the selection of the aforementioned process parameters was that previous studies found a strong correlation of these parameters with the mechanical strength of FFF parts [7]. The response parameters used for the present study were tensile strength (TS) in MPa, flexural strength (FS) in MPa and impact strength (IS) in MJ/m^2^. The primary motivation behind the selection of such response parameters was that FFF products withstand different types of loading conditions when used for automobile, aerospace and medical applications. Thus, it was obligatory to test each type of mechanical strength using the advanced optimization tool. These output parameters were taken as secondary data as reported by Panda et al. [24], and were designated as objective functions for the optimization study.

The objective functions are expressed by Equations (13)–(15) below:

(13) $$\mathrm{Maximize}\;\mathrm{TS}\;=\;13.5625\;+\;0.7156x_{1}\;-\;1.3123x_{2}\;+\;0.9760x_{3}\;+\;0.5183x_{5}\;+\;1.1671x_{1}^{2}\;-\;1.3014x_{2}^{2}\;-\;0.4363x_{1}x_{3}+\;0.4364x_{1}x_{4}\;-\;0.4364x_{1}x_{5}\;+\;0.4364x_{2}x_{3}\;+\;0.4898x_{2}x_{5}\;-\;0.5389x_{3}x_{4}\;+\;0.5389x_{3}x_{5}\;-\;0.5389x_{4}x_{5}$$

(14) $$\mathrm{Maximize}\;\mathrm{FS}\;=\;29.9178\;+\;0.8719x_{1}\;-\;4.8741x_{2}\;+\;2.4251x_{3}\;-\;0.9096x_{4}\;+\;1.6626x_{5}\;-\;1.7199x_{1}x_{3}\;+\;1.7412x_{1}x_{4}\;-\;1.1275x_{1}x_{5}\;+\;1.0621x_{2}x_{5}\;+\;1.0621x_{3}x_{5}+\;1.0408x_{4}x_{5}$$

(15) $$\mathrm{Maximize}\;\mathrm{IS}\;=\;0.401992\;+\;0.034198x_{1}\;+\;0.008356x_{2}\;+\;0.013673x_{3}\;+\;0.021383x_{1}^{2}\;+\;0.008077x_{2}x_{4}$$

The upper and lower limits of these parameters are defined as per machine constraints, and most commercial FFF printers are capable of fabricating products within the following parameter limits [24]. The parameter bounds are expressed by Equations (16)–(20).

(16) $$0.127\;\leq \;x_{1}\;\leq \;0.254$$

(17) $$0\;\leq \;x_{2}\;\leq \;30$$

(18) $$0\;\leq \;x_{3}\;\leq \;60$$

(19) $$0.4064\;\leq \;x_{4}\;\leq \;0.5064$$

(20) $$0\;\leq \;x_{5}\;\leq \;0.008$$

## 3. Results and Discussion

NNA was implemented in this work to solve the optimization problem of the FFF process. Because heuristic algorithms are stochastic optimization methods, to generate meaningful statistical results, they must be run more than 10 times at minimum.

Each simulation was carried out 30 times for this purpose, with population size varying from 20 to 40, and the maximum amount of iterations ranging from 100 to 500. Grey wolf optimization (GWO), novel bat algorithm (NBA), dragonfly algorithm (DA), salp swarm algorithm (SSA) and sine cosine algorithm (SCA) [25,26,27,28,29] were selected to check and compare the efficiency of NNA. The algorithm parameter settings used for comparison are shown in Table 1. The value of different parameters, such as the number of particles (NP), dimension size (D), maximum number of iterations (G_max_) and other algorithm variables (A, f_min_, f_max_, c1, c2, c3, *w*, s, c, e, α, γ and β) are shown in Table 1. It must be noted that for each of the algorithms, 20–40 search agents and 100–500 iterations for different setups were used.

The optimum solutions obtained by simulated algorithms are given in Table 2, Table 3 and Table 4 for TS, FS and IS estimation, respectively. It is evident from these outcomes that the optimum NNA’s fitness values are the same as that of competitive algorithms for TS, FS and IS fitness functions.

The performance of the simulated algorithms for FFF over 30 independent runs for 500 iterations and with a population size of 40 is given in Table 5, Table 6 and Table 7 for TS, FS and IS estimation, respectively. The results are presented in terms of best, worst, mean and standard deviation values over 30 runs.

**Figure 3:**
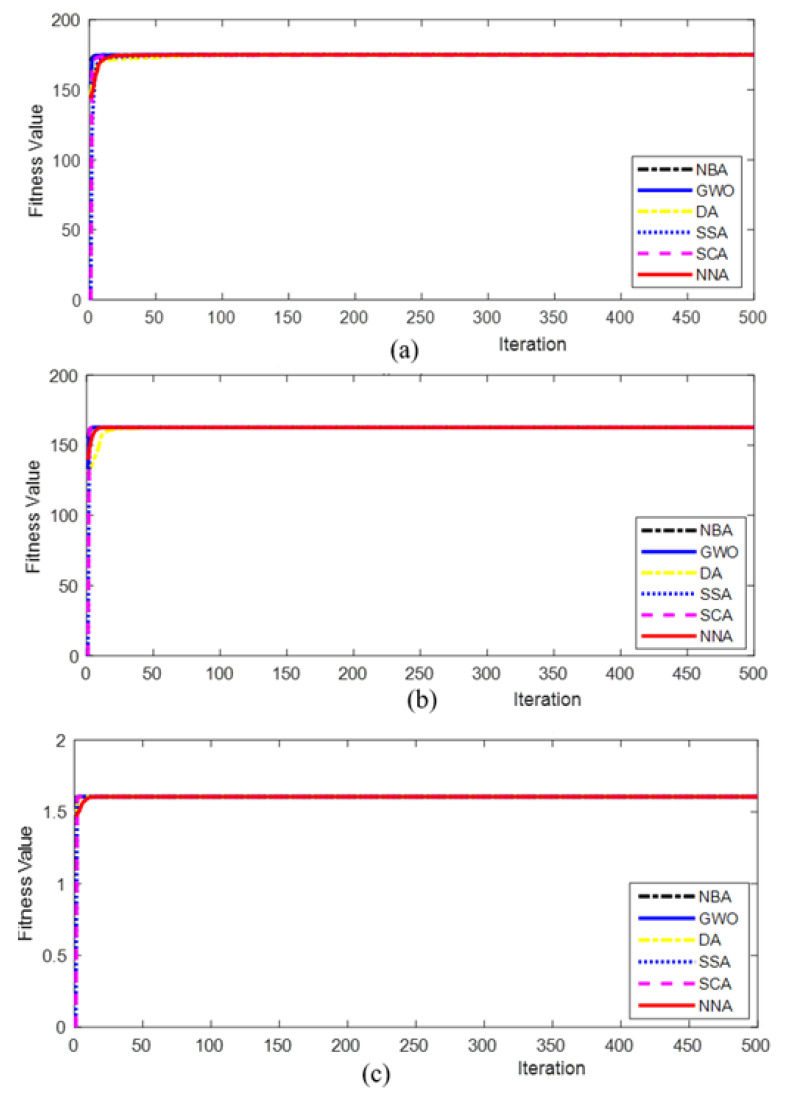
The convergence graph of simulated algorithm estimations over 30 independent runs for 500 iterations and population size 40 for (**a**) TS (**b**) FS and (**c**) IS.

The convergence rate of NNA, as shown in Figure 3, is also better than the competitive algorithms. The convergence characteristics for TS, FS and IS estimation are shown in Figure 3. It is found that for TS estimation, GWO, DA, SSA and NBA algorithms achieve optimum value, but overall, NNA has the capability to identify the best value with the least standard deviation. For FS estimation, GWO, SSA, SCA and NNA have the potential to reach global optimal solutions with the same standard deviation. For IS estimation, all algorithms are able to reach a near global optimal solution with the same standard deviation. Overall, the result demonstrates that NNA’s mean, median and standard deviation values are much better compared to competitive algorithms, which proves the enhanced exploration and exploitation capabilities of NNA for process parameter optimization of FFF, especially in TS estimation.

**Figure 4:**
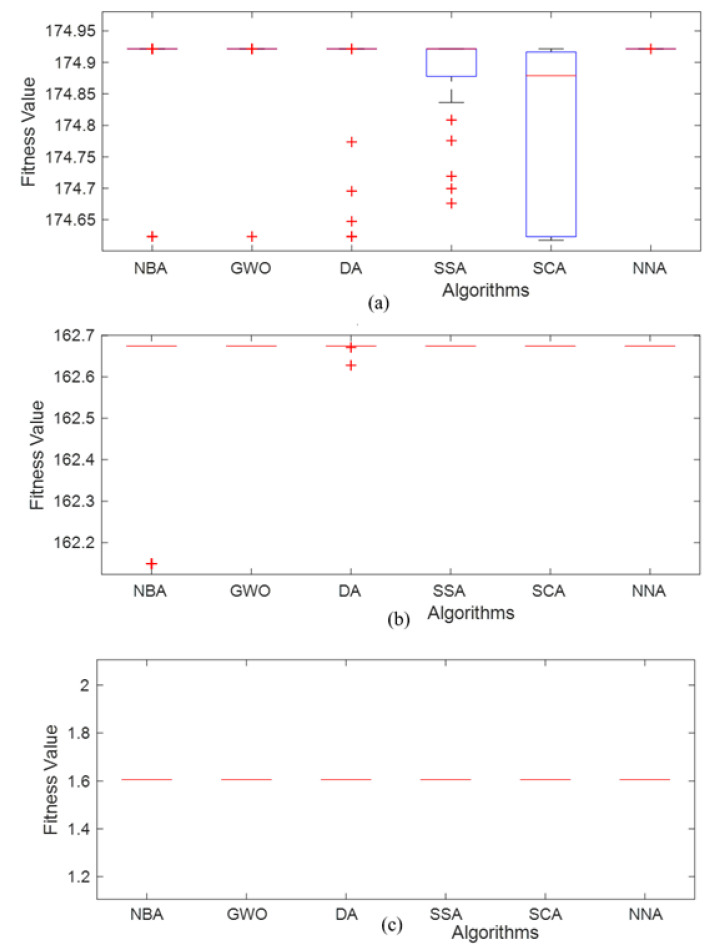
The box plot of simulated algorithm estimations over 30 independent runs for 500 iterations and population size 40 for (**a**) TS (**b**) FS and (**c**) IS.

The box plots for TS, FS and IS evaluations are displayed in Figure 4a–c, respectively, for NBA, GWO, DA, SSA, SCA and NNA. These box plots are an excellent medium to ascertain the fitness values and compare the effectiveness of different algorithms. For TS estimation, it is clear that NNA yields the best fitness values as mean and median, along with the least standard deviation. As compared to other rival algorithms, the NNA performs better, and these results can be used for the fabrication of commercial products using ABS. From the box plots of FS estimation, it is clear that GWO, SSA, SCA and NNA have similar mean and median fitness values, and their standard deviation values are better in comparison to NBA and DA. Thus, the overall performance of GWO, SSA, SCA and NNA is found to be better for FS estimation compared to other algorithms. From the box plots of IS estimation, it is clear that all optimization algorithms have similar mean and median fitness values, their standard deviation values are almost the same, and the algorithms reach optimum values.

Further, the performance comparison of metaheuristic algorithms for FFF over 30 independent runs for 100 iterations and population size 40 are given in Table A1, Table A2 and Table A3 (see Appendix A) for TS, FS and IS estimation, respectively. The effect of the reduced total number of iterations clearly shows that NNA’s mean, median and standard deviation values for TS estimation are much better compared to competitive algorithms, which proves that NNA is able to find an optimum solution with reduced iterations for process parameter optimization of FFF. Similarly, for FS estimation, SSA, SCA and NNA are able to achieve global optimum with equal authority. However, in IS estimation, each algorithm shows a similar effect to achieve the optimum value.

The performance of optimization algorithms for FFF process parameter estimation was evaluated over 30 independent runs for 100 iterations with population size 20. The results are given in Table A4, Table A5 and Table A6 (see Appendix A) for TS, FS and IS estimation, respectively, which demonstrate that NNA’s mean, median and standard deviation values are much better compared to competitive algorithms for TS and FS estimation. This proves the enhanced exploration and exploitation capabilities of NNA for the process parameter optimization of FFF. However, each algorithm is able to achieve the optimum value for IS estimation, independent of the population size of variables and the given number of iterations. The maximum values of TS, FS and IS can be achieved by manufacturing FFF parts using the process parameters predicted by the NNA algorithm.

The optimum parameter settings to achieve maximum tensile strength of FFF parts are layer thickness of 0.127 mm, 9.55° orientation angle, 60° raster angle, 0.4064 mm raster width and 0.008 mm air gap. On the other hand, flexural strength can be maximized at 0.127 mm layer thickness, 0° orientation angle, 60° raster angle, 0.4064 mm raster width and 0.008 mm air gap. In order to obtain the maximum value of impact strength, the FFF process must be carried out at a layer thickness of 0.254 mm, 30° orientation angle, 60° raster angle, 0.5064 mm raster width and 0.0079 mm air gap. It can be observed that a raster angle of 60° results in the maximization of all three parameters of mechanical strength, i.e., tensile, flexural and impact strength. Thus, it is recommended to use the default setting of 60° raster angle for the best mechanical stability of FFF parts. In the case of layer thickness, a lower value results in better impact and tensile strength, while the opposite phenomenon is predicted for flexural strength. The air gap is also found to be constant for mechanical stability. In the case of orientation angle, the horizontal layer deposition (0°) strategy is the most successful in attaining maximum flexural strength, while 9.55° and 30° result in better tensile and impact strength, respectively, as suggested by NNA.

## 4. Confirmatory Experiments

The optimum parameters for each output suggested by NNA must be validated before recommendation; hence, confirmatory experiments were conducted. The ABS samples were prepared using commercial P400 material (supplied by Stratasys Inc. Ltd., Eden Prairie, Minnesota, USA) and an open-source FFF printer (supplied by Prusa Research, Prague, Czech Republic). The technical data sheet [23] of thermoplastic materials was referred to when deciding printing parameters. The three samples were prepared for tensile strength, flexural strength and impact strength using ASTM D638, ASTM D790 and ASTM D256, respectively. The test samples were prepared at a constant printing speed, infill method, extrusion temperature, bed temperature and environmental temperature, as shown in Table 8.

**Figure 5:**
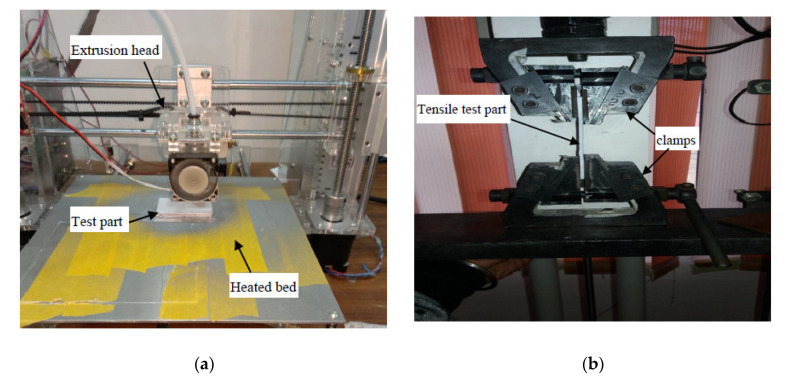
(**a**) FFF printer used for experimentation (**b**) Universal Testing Machine used for mechanical testing.

Fixed values of temperature were used to eliminate error as the significant impact of temperature on the bonding strength of ABS layers has been noticed. As the semi-molten thermoplastic beads are laid down, excessive temperature at the nozzle may increase flowability and cause deformation. On the other hand, faster cooling may lead to shrinkage, which would exhibit dimensional variation. Thus, the optimum values of temperature were selected as the thermoplastic polymer layers must be cooled slowly to avoid deformation and dimensional variability [19]. The printing and testing of samples was performed under controlled environmental conditions and using three replications of each experiment to avoid random errors (Figure 5).

The variable parameters used for fabrication and experimentation are shown in Table 9. The values of each parameter are taken from predictions made by NNA for tensile strength, flexural strength and impact strength. It can be observed that there is minimal variation between the predicted and experimental value of each output. The maximum variation of 2.92% occurs in sample no. 3, which undergoes impact testing, and the minimum variation (1.3%) occurs in sample no. 2 during flexural testing. The average variation of three experiments is 1.94%, which is much less compared to previous studies. Hence, the NNA predicted results are validated with high accuracy, meaning it is suitable for solving optimization issues in manufacturing processes.

**Figure 6:**
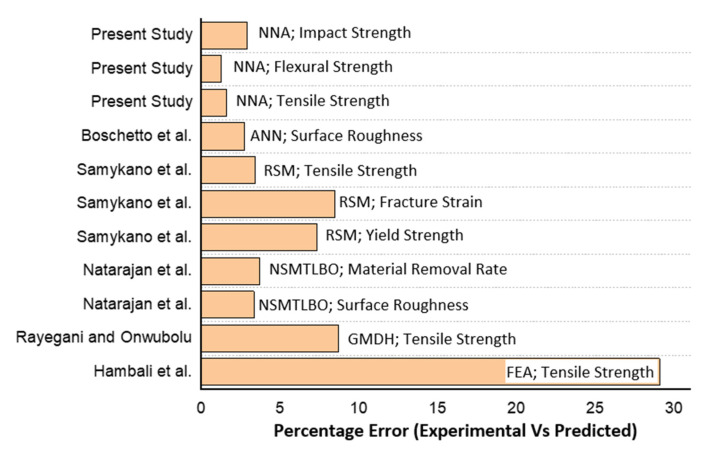
Comparison of percentage error of NNA results with previous studies.

The NNA results achieved in the present study are compared with the percentage error (experimental vs. predicted) of previous studies in Figure 6. The response parameter and prediction tool used by each author is also indicated in the graph. It can be observed that the minimum variation between experimental and predicted results is achieved in the present study as compared to previous studies that implemented advanced optimization algorithms. The maximum variation was found for finite element analysis (FEA) [2], while the average error was found using artificial neural networks (ANN) [30] and response surface methodology (RSM) [31].

The comparative analysis indicates the higher efficacy of NNA as compared to other optimization algorithms and prediction tools. The predicted parameters can be recommended for commercial production of functional prototypes and end-use components manufactured using FFF. Moreover, the NNA must be implemented to solve surface roughness and dimensional variability issues of FFF technology when used for complex designs.

## 5. Conclusions

Despite an efficient additive manufacturing technique, mechanical strength is one of the major obstructions against the applicability of FFF polymer products for end-use components. Thus, the selection of optimum parameter settings is required to obtain the best mechanical properties of ABS parts. The optimization was conducted employing NBA, GWO, DA, SSA, SCA and NNA algorithms for the maximization of mechanical strength (TS, FS and IS) of parts with varying population size and number of iterations. From the statistical and convergence results, it was found that the NNA algorithm was highly competitive with respect to NBA, GWO, DA, SSA and SCA algorithms and was able to achieve optimum results for FFF products. The endorsed input parameters indicated the manifestation of enhanced mechanical strength among the test parts. In the future, researchers can also implement a hybrid metaheuristic algorithm for the enhancement of quality and accuracy along with a reduction in convergence time.

## Figures and Tables

**Table 1:** Parameter settings for different algorithms.

Algorithm	Parameters
NBA	*NP* = 20–40; *D* = 5; *G_max_* = 100–500; A = 0.5; r = 0.5; α = γ = 0.9; $f_{min}=0;f_{max}=1.5$
GWO	*NP* = 20–40; *D* = 5; *G_max_* = 100–500; *a =* [0,1,2]
DA SSA SCA	*NP* = 20–40; *D* = 5; *G_max_* = 100–500; *w =* [0.4–0.9], *s =* 0.1, *a* = 0.1, *c* = 0.7, *f* = 1, *e* = 1 *NP* = 20–40; *D* = 5; *G_max_* = 100–500; c1, c2, c3 = [0,1] *NP* = 20–40; *D* = 5; *G_max_* = 100–500; *a =* 2
NNA	*NP* = 20–40; *D* = 5; *G_max_* = 100–500; $\beta_{NNA}$ = [1–0.05]; $\alpha_{NNA}$ = 0.99

**Table 2:** Optimum solutions obtained by optimization algorithms for tensile strength (TS) estimation for 500 iterations with population size 40.

Process Parameters	Units	NBA	GWO	DA	SSA	SCA	NNA
*x_1_*	mm	0.127	0.127	0.127	0.127	0.127	0.127
*x_2_*	degrees	9.557223075	9.556302041	9.555264322	9.554816312	9.418607509	9.557224815
*x_3_*	degrees	60	60	60	60	60	60
*x_4_*	mm	0.4064	0.4064	0.4064	0.4064	0.4064	0.4064
*x_5_*	mm	0.008	0.008	0.008	0.008	0.008	0.008
*y*	---	1.605823622	1.605823622	1.605823622	1.605823622	1.605823622	1.605823622

**Table 3:** Optimum solutions obtained by optimization algorithms for flexural strength (FS) estimation for 500 iterations with population size 40.

Process Parameters	Units	NBA	GWO	DA	SSA	SCA	NNA
*x_1_*	mm	0.127	0.127	0.127	0.127	0.127	0.127
*x_2_*	degrees	0	0	0	0	0	0
*x_3_*	degrees	60	60	60	60	60	60
*x_4_*	mm	0.4064	0.4064	0.4064	0.4064	0.4064	0.4064
*x_5_*	mm	0.008	0.008	0.008	0.008	0.008	0.008
*y*	-	162.6744472	162.6744472	162.6744472	162.6744472	162.6744472	162.6744472

**Table 4:** Optimum solutions obtained by optimization algorithms for impact strength (IS) estimation for 500 iterations with population size 40.

Process Parameters	Units	NBA	GWO	DA	SSA	SCA	NNA
*x_1_*	mm	0.254	0.254	0.254	0.254	0.254	0.254
*x_2_*	degrees	30	30	30	30	30	30
*x_3_*	degrees	60	60	60	60	60	60
*x_4_*	mm	0.5064	0.5064	0.5064	0.5064	0.5064	0.5064
*x_5_*	mm	0.007951741	0.007951741	0.007951741	0.007951741	0.007951741	0.007951741
*y*	-	1.605823622	1.605823622	1.605823622	1.605823622	1.605823622	1.605823622

**Table 5:** The performance of simulated algorithms for TS estimation over 30 independent runs for 500 iterations and population size 40.

Algorithm	Worst	Best	Average	Median	Std. Dev.
NBA	174.6234301	174.9214992	174.9016279	174.9214992	7.56 × 10^−2^
GWO	174.6234298	174.9214992	174.911563	174.9214991	5.44 × 10^−2^
DA	174.6234301	174.9214992	174.8800297	174.9214992	9.72 × 10^−2^
SSA	174.6761253	174.9214992	174.8844652	174.9214992	7.32 × 10^−2^
SCA	174.6177085	174.9214213	174.7818994	174.8788781	1.44 × 10^−1^
NNA	174.9214992	174.9214992	174.9214992	174.9214992	1.89 × 10^−10^

**Table 6:** The performance of simulated algorithms for FS estimation over 30 independent runs for 500 iterations and population size 40.

Algorithm	Worst	Best	Average	Median	Std. Dev.
NBA	162.1491001	162.6744472	162.6044009	162.6744472	1.82 × 10^−1^
GWO	162.6744472	162.6744472	162.6744472	162.6744472	5.78 × 10^−15^
DA	162.6278193	162.6744472	162.6727806	162.6744472	8.51 × 10^−3^
SSA	162.6744472	162.6744472	162.6744472	162.6744472	5.78 × 10^−14^
SCA	162.6744472	162.6744472	162.6744472	162.6744472	5.78 × 10^−14^
NNA	162.6744472	162.6744472	162.6744472	162.6744472	5.78 × 10^−14^

**Table 7:** The performance of simulated algorithms for IS estimation over 30 independent runs for 500 iterations and population size 40.

Algorithm	Worst	Best	Average	Median	Std. Dev.
NBA	1.605823622	1.605823622	1.605823622	1.605823622	2.26 × 10^−16^
GWO	1.605823622	1.605823622	1.605823622	1.605823622	2.26 × 10^−16^
DA	1.605823622	1.605823622	1.605823622	1.605823622	2.26 × 10^−16^
SSA	1.605823622	1.605823622	1.605823622	1.605823622	2.26 × 10^−16^
SCA	1.605823622	1.605823622	1.605823622	1.605823622	2.26 × 10^−16^
NNA	1.605823622	1.605823622	1.605823622	1.605823622	2.26 × 10^−16^

**Table 8:** Fixed parameters used for confirmatory experiments.

S. No.	Fixed Parameter	Value
1	Extrusion temperature	230 °C
2	Bed temperature	80 °C
3	Ambient temperature	25 °C
4	Infill type	Rectilinear
5	Printing speed	50 mm/s

**Table 9:** Variable parameters and results.

Sample No.	Layer Thickness (mm)	Orientation Angle (°)	Raster Angle (°)	Raster Width (mm)	Air Gap (mm)	Predicted Value	Experimental Value	Error (%)
1	0.127	9.55	60	0.4064	0.008	17.49	17.23	1.61
2	0.127	0	60	0.4064	0.008	16.26	16.05	1.3
3	0.254	30	60	0.5064	0.0079	2.46	2.39	2.92

## Appendix A

**Table A1:** The performance of simulated algorithms for TS estimation over 30 independent runs for 100 iterations and population size 40.

Algorithm	Worst	Best	Average	Median	Std. Dev.
NBA	174.6234301	174.9214992	174.9011405	174.921458	7.52 × 10^−2^
GWO	174.623429	174.9214992	174.8620095	174.9214386	1.05 × 10^−1^
DA	174.6234301	174.9214992	174.8394289	174.9201719	1.18 × 10^−1^
SSA	174.6324965	174.9214992	174.8028863	174.8169921	9.99 × 10^−2^
SCA	174.4505185	174.9200412	174.6821489	174.6282199	1.24 × 10^−1^
NNA	174.8553077	174.9214939	174.9100097	174.9200154	1.98 × 10^−2^

**Table A2:** The performance of simulated algorithms for FS estimation over 30 independent runs for 100 iterations and population size 40.

Algorithm	Worst	Best	Average	Median	Std. Dev.
NBA	162.1491001	162.6744472	162.5693778	162.6744472	2.14 × 10^−1^
GWO	162.674447	162.6744472	162.6744472	162.6744472	3.38 × 10^−8^
DA	162.6627456	162.6744472	162.6740571	162.6744472	2.14 × 10^−3^
SSA	162.6744472	162.6744472	162.6744472	162.6744472	5.78 × 10^−14^
SCA	162.6744472	162.6744472	162.6744472	162.6744472	5.78 × 10^−14^
NNA	162.6744472	162.6744472	162.6744472	162.6744472	5.78 × 10^−14^

**Table A3:** The performance of simulated algorithms for IS estimation over 30 independent runs for 100 iterations and population size 40.

Algorithm	Worst	Best	Average	Median	Std. Dev.
NBA	1.605823622	1.605823622	1.605823622	1.605823622	2.26 × 10^−16^
GWO	1.605823622	1.605823622	1.605823622	1.605823622	2.26 × 10^−16^
DA	1.605823622	1.605823622	1.605823622	1.605823622	2.26 × 10^−16^
SSA	1.605823622	1.605823622	1.605823622	1.605823622	2.26 × 10^−16^
SCA	1.605823622	1.605823622	1.605823622	1.605823622	2.26 × 10^−16^
NNA	1.605823622	1.605823622	1.605823622	1.605823622	2.26 × 10^−16^

**Table A4:** The performance of simulated algorithms for TS estimation over 30 independent runs for 100 iterations and population size 20.

Algorithm	Worst	Best	Average	Median	Std. Dev.
NBA	174.6234263	174.9214936	174.8372247	174.9196852	1.27 × 10^−1^
GWO	174.6242344	174.921498	174.8359942	174.9200114	1.11 × 10^−1^
DA	171.631808	174.9214992	174.1513635	174.6316391	1.04
SSA	174.5267321	174.9214992	174.7720952	174.7517588	1.25 × 10^−1^
SCA	174.1631947	174.921241	174.5762605	174.5885636	2.10 × 10^−1^
NNA	174.6348368	174.9214885	174.8893958	174.9163291	6.93 × 10^−2^

**Table A5:** The performance of simulated algorithms for FS estimation over 30 independent runs for 100 iterations and population size 20.

Algorithm	Worst	Best	Average	Median	Std. Dev.
NBA	162.1491001	162.6744472	162.6044009	162.6744472	1.82 × 10^−1^
GWO	162.6734733	162.6744472	162.6743693	162.6744472	2.15 × 10^−4^
DA	162.4635226	162.6744472	162.6453716	162.6744472	4.66 × 10^−2^
SSA	162.6744472	162.6744472	162.6744472	162.6744472	5.78 × 10^−14^
SCA	162.1491001	162.6744472	162.6569356	162.6744472	9.59 × 10^−2^
NNA	162.6744472	162.6744472	162.6744472	162.6744472	5.78 × 10^−14^

**Table A6:** The performance of simulated algorithms for IS estimation over 30 independent runs for 100 iterations and population size 20.

Algorithm	Worst	Best	Average	Median	Std. Dev.
NBA	1.605823622	1.605823622	1.605823622	1.605823622	2.26 × 10^−16^
GWO	1.605823622	1.605823622	1.605823622	1.605823622	2.26 × 10^−16^
DA	1.605823622	1.605823622	1.605823622	1.605823622	2.26 × 10^−16^
SSA	1.605823622	1.605823622	1.605823622	1.605823622	2.26 × 10^−16^
SCA	1.605823622	1.605823622	1.605823622	1.605823622	2.26 × 10^−16^
NNA	1.605823622	1.605823622	1.605823622	1.605823622	2.26 × 10^−16^
